# Omega-3 Fatty Acid Blood Levels Clinical Significance Update

**DOI:** 10.1007/s12170-014-0407-4

**Published:** 2014-09-26

**Authors:** H. Robert Superko, Alex R. Superko, Gina P. Lundberg, Basil Margolis, Brenda C. Garrett, Khurram Nasir, Arthur S. Agatston

**Affiliations:** 1 Cholesterol, Genetics, Heart Disease Institute, Mercer University School of Pharmaceutical Sciences, 40 Bear Paw, Portola Valley, CA 94028 USA; 2 M3 Environmental, 1820 Vallejo St, Seaside, CA 93955 USA; 3 Emory Saint Joseph’s Hospital, Emory University School of Medicine, 5673 Peachtree Dunwoody Rd, Atlanta, GA 30342 USA; 4 Cholesterol, Genetics, Heart Disease Institute, 40 Bear Paw, Portola Valley, CA USA; 5 Baptist Healthcare System, Florida State University School of Medicine, 1691 Michigan Ave. Suite 500, Miami, FL 33139 USA

**Keywords:** Fish oil, Eicosapentaenoic acid, Docosahexaenoic acid, EPA, DHA, Cardiovascular disease, Heart disease, Myocardial infarction, Sudden death, Fatty acid, n-3 fatty acid, Omega-3, Blood measurement, Polyunsaturated fatty acid, n-3 PUFA, Population distribution, Coronary artery calcium

## Abstract

The potential benefit of fish oil (omega-3 fatty acids) consumption to reduce cardiovascular disease (CVD) risk remains controversial. Some investigations report reduced CVD risk associated with fish or fish oil consumption while others report no benefit. This controversy is in part resolved when consideration is given to omega-3 blood levels in relation to CVD risk as well as blood levels achieved in clinical trials of omega-3 supplementation and CVD benefit. There is a wide variation in omega-3 blood levels achieved between individuals in response to a given dose of an omega-3 supplement. Many studies tested a daily dose of 1 gram omega-3 supplementation. The individual variation in blood omega-3 levels achieved in response to a fixed daily dose helps to explain why some individuals may obtain CVD protection benefit while others do not due to failure to achieve a therapeutic threshold. Recent development of a population range in a United States population helps to provide clinical guidance since population omega-3 blood level ranges may vary due to environmental and genetic reasons. Omega-3 supplementation may also be of benefit in reducing the adverse impact of air pollution on CVD risk.

## Intoduction

The new AHA Cholesterol guidelines suggest determining statin treatment strategy based on the risk classification of the patient [1]. While this is a commendable approach, the approximately 25 % relative coronary heart disease (CHD) risk reduction attributed to statin treatment is actually only an approximate 3.4 % absolute risk reduction. This approach leaves many people still at elevated CHD risk despite statin therapy [2]. Blood fish oil levels appears to reflect CHD risk as well as clinical benefit following omega-3 treatment that may be of clinical utility in the large group of patients that remain at CHD risk despite statin treatment.

Probably the first report of fish consumption as a medical therapy is found in the Old Testament book of Tobias, “*Then the angel said to him*: *Take out the entrails of the fish*, *and lay up his heart*, *and his gall*, *and his liver for thee*; *for these are necessary for useful medicines*.“ [3]. In relatively recent times multiple studies have confirmed this observation. Large studies such as the Swedish Mammography cohort of 36,234 women followed for 8 years suggests moderate consumption of fatty fish is associated with a lower risk for heart failure hospitalization [4]. However, clinical trials of dietary modification or fish oil consumption have not consistently reported significant cardiovascular disease (CVD) event reduction from fish oil intake. Part of the confusion surrounding the different conclusions of these studies involves the assessment of the effect of either a fixed daily dose of omega-3, or the measurement of omega-3 blood levels achieved in each patient. The controversy regarding daily dose of fish oil versus achieved blood level has recently been reviewed [5•]. This is an important issue since wide variability in individual blood level achieved, in response to a fixed dose of fish oil, has been reported [5•]. In response to a fixed dose of fish oil, some patients may achieve a therapeutic level while others may not. In the past year new reports have contributed to the clarification of this topic. The purpose of this article is to place recent reports into clinical context regarding blood levels of omega-3 fatty acids and CVD risk or benefit.

Omega-3 fatty acid blood level measurement can be performed in either plasma, serum, or red blood cell membranes. Measurements with clinical utility include the “Omega-3 Index” which is the percent of fatty acids composed of eicosapentaenoic acid (EPA) + docosahexaenoic acid (DHA), the EPA/AA (arachadonic acid) ratio, EPA quantitation, and DHA quantitation. Several laboratory methods are available and include gas chromatography, red blood cell membrane composition, and mass spectroscopy [6–8].

## Controversey Remains

The controversy over potential CVD benefit of omega-3 fatty acids has not been resolved in the past two years. A recent meta-analysis of 16,338 subjects treated with omega-3 fatty acids and 16,318 control subjects did not demonstrate satisfactory improvements in major cardiovascular events but omega-3 blood levels were not part of the analysis [9]. However, in the same analyses, significant reductions in risk of death from cardiac causes was demonstrated reflecting a reduced CVD mortality benefit from omega-3 supplementation. The Singapore Chinese Health Study utilized a semi-quantitative food-frequency questionnaire together with mortality information in 63,257 Chinese adults with 890,473 person-years of follow up. Both EPA, DHA, and ALA intake estimated from the food frequency questionnaire were independently associated with reduced risk of CV mortality [10]. The Spanish EPIC cohort study also estimated omega-3 fatty acid dietary intake from a dietary questionnaire [11]. Contrary to the Singapore study, the Spanish EPIC cohort did not reveal any association of estimated EPA and DHA intake to incident CHD in either men or women but neither study assessed blood levels of omega-3 fatty acids achieved in individual patients. Two recent 1 gram/day omega-3 supplement studies have reported no CV event benefit after 1 year and 6.2 years of follow up [12, 13]. However similarly, neither of these studies investigated the blood levels of omega-3 fatty acids achieved in individual subjects. This is an important difference in study design since an investigation such as the Multi-Ethnic Study of Atherosclerosis (MESA), which did measure blood levels of omega-3 fatty acids, has reported that circulating EPA and DHA blood levels were inversely and significantly associated with incident CVD with a hazard ratio of 0.49 for the highest quartile of blood EPA level and 0.39 for the highest quartile of blood DHA, compared with the lowest quartile [14]. No significant associations with CVD were observed for alpha-linolenic acid or n-6 PUFA. This may be of particular relevance due to the use of a 1 gram/day omega-3 dose which may leave many patients below a therapeutic threshold. Finally, part of the confusion may involve the effect of omega-3 supplementation in patients based on the presence of other CHD risk factors. The JELIS study reports that a 39 % (p = 0.007) reduction in major coronary events was primarily seen in patients who did not achieve LDL-C and non HDL-C goals and concluded that EPA supplementation may be most useful for those patients who cannot achieve blood lipid goals [15].

The controversy regarding daily dose of omega-3 versus individual blood levels achieved is further complicated by including the concept of replacing dietary saturated fat with omega-6 linoleic acid (LA). LA is found in high concentrations in safflower oil, sunflower oil, cottonseed oil, corn oil, and soybean oil. Initially recommended as replacements for saturated fat in the diet, the findings of the Sydney Diet Heart Study suggests this may be a mistake [16]. The Sydney Diet Heart Study was a randomized controlled trial in 458 men conducted between 1966 to 1973. The intervention group received replacement of dietary fat with omega-6 linoleic acid in the form of safflower oil and polyunsaturated margarine and had higher rates of death than controls (17.6 % versus 11.8 %, hazard ratio 1.62) suggesting that substituting dietary linoleic acid in place of saturated fats actually increased the rates of death from all causes, CHD, and CVD. Thus, deleterious effects from LA included in omega-3 supplements may substantially attenuate a benefit from omega-3 supplementation.

## Variability in Individual Omega-3 Blood Level Response to Treatment

Many fish oil clinical trials have used a dose of 1 g/d in all treated subjects. The dose of 1 g/d of EPA, has been reported to increase the total EPA + DHA blood levels from a mean of 3.6 % to 5.4 % but approximately 16 % of the subjects achieved an EPA + DHA blood level less than 4.8 % [17]. This is clinically relevant since it has been noted that an EPA + DHA blood level > 5 % is the range in which dramatic reduction in sudden coronary death reduction can be observed [17]. Individual variability in blood level response to such a common dose could leave a substantial number of patients at elevated CHD risk due to failure to achieve a therapeutic EPA + DHA blood level. The blood EPA/AA ratio is also a clinically relevant measurement and has substantial individual variability in response to a fixed dose. An EPA/AA ratio >0.75 has been associated with significantly lower major coronary events (MCE) in a Japanese population [18•]. Laidlaw and colleagues have reported that 4 g/d of fish oil increased the mean EPA/AA ratio from approximately 0.12 to 0.9 [19]. It is estimated that approximately 68 % of the subjects would have obtain an EPA/AA ratio of between 0.78 and 1.02 while 16 % would be less than 0.78. Thus, even with 4 g/d omega-3 supplementation, approximately 16 % of subjects would not have achieved the putative EPA/AA goal.

A portion of this variability is related to genetic differences in fatty acid metabolism [20]. The ability to predict omega-3 index RBC response to therapy also depends on simple measurements such as body weight. Flock and colleagues investigated the individual omega-3 index response to 0, 300, 600, 900, and 1800 mg/d of EPA + DHA supplementation and reported that adding weight to the prediction model containing EPA + DHA dosing changed predictive power of the response variability from 68 % to 70 % (p < 0.0001) and adding additional factors such as baseline omega-3 index, age, gender, and physical activity further improved power of prediction of treatment response to 78 % [21•].

## Population Ranges

Blood omega-3 levels may vary for a variety of lifestyle (e.g. fish consumption), geographic, and genetic reasons. In Japan the mean EPA/AA ratio in patients requiring PCI was 0.40 and in an asymptomatic population was 0.41 [22, 23•]. In these two Japanese studies, both reported significant association between EPA/AA ratio and coronary events despite the fact that one enrolled patients with prior coronary event and the other enrolled apparently health patients.

The difference is even more pronounced between different ethnic groups. Mean and standard deviation values from Caucasian individuals with sudden cardiac death compared to a control group in the Physicians’ Health Study, revealed a mean ± SD (%) EPA of 1.72 ± 0.59 and DHA 2.12 ± 0.65 in the sudden death group and EPA of 1.84 ± 0.53 and DHA 2.38 ± 0.78 in the control group [24]. This reflects a mean omega-3 index of 3.84 % in the sudden death group compared to 4.22 % in the control group. For the EPA/AA ratio, in a Japanese population the mean ± SD is reported to be 0.63 ± 0.40 [25].

**Fig. 1:**
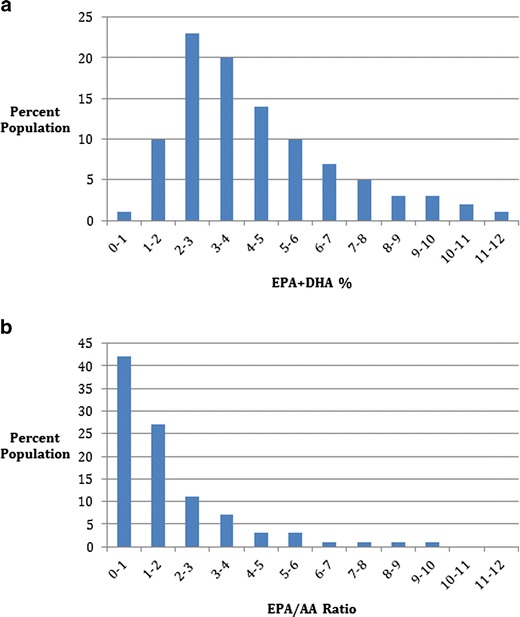
**a** Population distribution EPA + DHA % in 1,101 subjects. Mean = 4.42 %, SD = 2.59, median = 3.7 %. **b** Population distribution EPA/AA ratio in 1,097 subjects. Mean = 1.96, SD = 2.61, median = 1.0

For individual patient values to be of clinical use it is often useful to compare them to a reference range that is determined from a population of the same nationality due to variability in diet and environmental issues, and utilizing the same laboratory analysis method as the one utilized for patient management. Many of the studies previously referred to were conducted in Europe or Asia where variation in dietary and other conditions may have resulted in omega-3 blood level ranges different from those in the United States. For this reason a reference range study was conducted in 1,101 healthy male and female adults residing in the Los Angeles region to determine laboratory reference ranges. Figure 1a represents the omega-3 index reference range. For the omega-3 index, the 25th percentile was 2.5 %, the 50th percentile was 3.7 %, and the 75th percentile was 5.7 %. Figure 1b represents the EPA/AA ratio in this healthy USA population and reveals a 25th percentile of 0.6, a 50th percentile of 1.0, and a 75th percentile of 2.3. The mean and SD was 1.96 ± 2.61.

## EPA/AA Ratio as a Therapeutic Goal

The blood EPA/AA ratio is a clinically relevant measurement. An EPA/AA ratio >0.75 has been associated with significantly lower major coronary events (MCE) in a Japanese population [26]. The EPA/AA ratio was also reported to have a linear relationship with the ratio of prostaglandin (PG) I3 and PGI_2_ to thromboxane (TXA_2_) [27]. Most recently, the Hisayama Study evaluated the EPA/AA ratio in 3,103 apparently healthy Japanese adults and reported significantly increased incidence rates of CVD with lower EPA/AA ratios [23•]. This was particularly evident in subjects with hs-CRP ≥ 1.0 mg/L but not in those with hs-CRP < 1.0 mg/L. In subjects with an EPA/AA ratio <0.29 but a hs-CRP ≥ 1.0 mg/L the age and sex adjusted incidence (per thousand person years) was 23.3 compared to subjects with the same EPA/AA ratio but hs-CRP < 1.0 mg/L which was 8.2. The CVD risk increased 1.52 times per 0.20 decrement in serum EPA/AA ratio in those with hs-CRP ≥ 1.0 mg/L. This association was not found for stroke in the Hisayama study. In contrast, in Japanese patients with acute ischemic stroke, early neurological deterioration (END) was reported to be significantly associated with diabetes mellitus, hs-CRP, prior stroke, ischaemic heart disease, small vessel disease, and the EPA/AA and DHA/AA ratios [28]. The investigators concluded that a low serum n-3 PUFA/n-6 PUFA ratio on admission may predict neurological deterioration with acute ischemic stroke. The role of omega-3 fatty acids in patients undergoing percutaneous coronary intervention (PCI) remains a topic of interest but has a paucity of information. Domei and colleagues recently reported on 284 PCI patients with major adverse cardiac events (MACE) associated with the PCI [22]. Analysis of serum omega-3 levels revealed that only a high serum EPA/AA ratio was significantly associated with a low incidence of MACE in multiple statistical models. A high level was defined as > 0.404. This remained significant after adjustment for age, diabetes, gender, hypertension, LDL-C, and smoking.

## Renal Disease

Maintenance hemodialysis therapy may impact omega-3 blood levels and CHD risk. In 517 patients, treated in Japan, EPA/AA and DHA/AA ratios were significantly reduced when compared to controls. Following adjustment for age and other variables, the patients with the lowest versus highest EPA + DHA/AA ratios exhibited HRs in the range of 1.7 to 2.0 [29•]. Interestingly, the absolute EPA, DHA, and AA concentrations were not predictive of CVD. The authors concluded that in hemodialysis patients, the serum PUFA profile is unfavorably altered and that the low n-3 PUFA/AA ratios are independently predictors of CVD.

In IgA Nephropathy patients, 2 years of treatment with 3.35 g/d total omega-3 fatty acids EPA levels increased from 0.8 ± 0.5 to 3.1 ± 1.3 % after treatment and DHA from 3.7 ± 1.6 to 6.5 ± 1.3 %. Treatment with 6.70 g/d of omega-3 fatty acids increased blood EPA levels from 0.9 ± 0.6 to 5.2 ± 1.8 % and blood DHA from 3.5 ± 1.4 to 7.1 ± 1.6 % [30]. In patients with IgA nephropathy, treated with 4 gm/d fish oil, the mean EPA/AA ratio increased from approximately 0.09 ± 0.07 to 0.45 ± 0.40 with a wide range of individual variability [30–32].

## Coronary Calcification

The Rotterdam Study reported that fish consumption was correlated with coronary calcium scores [33]. In the Honolulu Heart Study, Japanese living in Japan, had two-fold higher blood levels of omega-3 fatty acids than both Caucasian Americans living in Pennsylvania and Japanese Americans living in Honolulu [34•]. The prevalence of CAC in Japanese living in Japan was 9.3 %, in Caucasian Americans, 26.1 %, and in Japanese Americans 31.4 %. The highest median EPA + DHA% was associated with the lowest CAC prevalence .While significant differences in CAC prevalence between Japanese living in Japan and Caucasians living in America was present, these differences became non-significant after adjusting for serum omega-3 fatty acid levels. A recent investigation by Sekikawa and colleagues reported on a follow-up study of 214 Japanese men and 152 Caucasian men who initially had a calcium score of 0. After adjusting for age, systolic blood pressure, LDL-C, diabetes the Japanese men had a significantly lower incidence rate of CAC > 10 compared to Caucasian men [34•]. After adjusting for omega-3 fatty acids the ratio was attenuated and became non-significant. The authors concluded that omega-3 fatty acids significantly contribute to the difference in the incidence of CAC between Japanese and Caucasian men.

## Psychiatric and Depressive Disorders

Depression and psychiatric disorders have gained attention in regard to CVD risk and outcomes. Depression following myocardial infarction or cardiovascular procedures appears to contribute to future cardiovascular events [35]. Fatty acids are key components of normal brain function and omega-3 fatty acids may be important in this regard. A retrospective study of Japanese patients with acute ischaemic stroke and early neurological deterioration reported that blood levels of EPA/AA and DHA/AA were significantly and inversely associated with early neurological deterioration [28].

## Cancer Concern

The association of fish oil and prostate cancer dates back to at least 1994 [36]. Environmental factors appear to play a role in the development of hormone dependent cancers such as breast and prostate. Dietary fat intake has been widely studied in regard to risk for breast and prostate cancer but no definitive conclusion has been developed. In-vitro and animal studies indicate a protective effect of omega-3 fatty acids on the progression of tumors [37]. Following an extensive review of the epidemiologic data Terry and colleagues conclude that given a general lack of studies that included important measurements, such as type of fish consumed and tissue concentrations of omega-3 fatty acids, there are too few studies to conclude that a relationship exists between marine fatty acid intake and human cancer [38]. However, in 2011 Brasky and colleagues reported, in 1,658 cases and 1,803 controls, that serum levels of DHA (highest vs lowest quartile) were associated with biopsy determined high-grade prostate cancer but no fatty acids were associated with low-grade prostate cancer [39].

In 2013 Chua and colleagues published a literature search of 12 studies and concluded that high serum levels of DPA were associated with reduced total prostate cancer risk and that high levels of EPA and DHA could possibly be associated with increased risk [40]. Like-wise, Sorongon-Legaspi and colleagues published a meta-analysis of 6 case control and 6 nested case control studies also concluded that blood DPA had an inverse relationship to prostate cancer risk and that EPA and DHA had a positive association with high-grade prostate cancer risk but only after adjustment of inter-study variability, but advised caution in interpretation of the results [41]. Also in 2013 Torfadottir and colleagues published a report in 2,268 men using reported fish consumption but no blood measurements [42]. They concluded that salted or smoked fish may increase the risk of advanced prostate cancer whereas fish oil consumption may be protective against progression of prostate cancer in elderly men. In those with very high fish consumption no association was found between fish consumption, early or midlife, and prostate cancer risk.

Finally, Brasky and colleagues most recent report in 2013 generated considerable interest and is derived from the Selenium and Vit E Cancer Prevention Trial. This study reported an association of higher plasma linoleic acid with reduced risk of low-grade and total prostate cancer and that long chain fatty acids like EPA and DHA were associated with increased risk in this matched case control study [43].

## Environmental Effects

Some adverse environmental effects may be impacted by fish oils. Air pollution, and its adverse health consequences, is of growing concern and studies have indicated that exposure to ozone or air pollution particulate matter may increase angina or CHD events [44, 45]. Low heart rate variability is an independent predictor of sudden cardiac death, myocardial infarction, heart failure and arrythmias in CHD patients [46]. An effect on heart rate variability (HRV) of 2 g/d fish oil supplementation was reported in nursing home residents exposed to ambient air pollution in Mexico City. Omega-3 supplementation reduced HRV lowering by air pollution from −54 % pre-supplementation to −7 % post omega-3 supplementation (p < 0.01) [47]. This observation was tested in an experimental chamber study. A randomized, double blind study of Tong and colleagues investigated the potential protective effects of 3 gm/day omega-3 fatty acids when healthy middle aged adults were exposed to 2 hours of air pollution in an experimental chamber [45]. This study revealed that exposure to concentrated ambient fine and ultrafine particulate matter (CAP) induced acute cardiac and lipid changes that included significant elevations in triglycerides and VLDL-C as well as lowering of heart rate variability and QT interval changes. These parameters were not improved with olive oil supplementation but were mitigated by 3 gm/d of marine derived omega-3 fatty acids.

## Conclusions

The controversy regarding the clinical role of enhanced fatty fish consumption or omega-3 supplementation is gradually becoming clear but some controversy remains. It is becoming clear that blood levels of omega-3 fatty acids exhibit a relationship to CVD while fixed daily doses may not. First, as reported in the JELIS investigation, the benefit of fish oil consumption may be impacted by other risk factors such as LDL-C and non-HDL-C levels. Second, recent reports such as the MESA investigation confirm that reported circulating blood levels of EPA and DHA are inversely and significantly associated with reduced CHD event risk [14]. This supports the interpretation that the blood levels of omega-3 fatty acids achieved are more related to CVD benefit than the daily dose of fish oil supplements [5•]. Third, in order to determine the risk status of a patient, it is useful to have population ranges in the country in which the patient resides, and many studies have been conducted in either Japan or European countries. A recent investigation of 1,102 healthy men and women living in the Los Angeles region provides population ranges based on a US population. Fourth, much like the TC/HDL-C ratio provided a modestly improved CHD risk prediction ability, the EPA/AA ratio also appears to provide clinical utility. This appears to be of particular importance and the predictive ability enhanced when other factors, such as elevated hs-CRP, are present. Fifth, hemodialysis and nephropathy patients appear to be a group of patients that may benefit from omega-3 blood level testing but have a wide range of individual variability in regard to blood level response to an omega-3 dose. Sixth, noninvasive CAC testing utilized for clinical purposes of risk prediction and differences in CAC prevalence between Japanese and United States individuals appears to be linked to differences in omega-3 fatty acid blood levels. Seventh, omega-3 blood levels may be associated with neurological deterioration in ischemic stroke patients.

Research into the potential benefits of omega-3 fatty acids will continue and should include fatty acid blood measurements in individual patients. Currently clinical trial evidence exists that provides physicians information on individual patient risk classification and anticipated response to treatment that may be of clinical value. Physician clinical judgment remains an important element of excellent medical care and the fish oil controversy is no exception to this rule.
